# Effectiveness of Naltrexone in the Prevention of Delayed Respiratory Arrest in Opioid-Naive Methadone-Intoxicated Patients

**DOI:** 10.1155/2013/903172

**Published:** 2013-09-09

**Authors:** Abbas Aghabiklooei, Hossein Hassanian-Moghaddam, Nasim Zamani, Shahin Shadnia, Mohammad Mashayekhian, Mitra Rahimi, Soheil Nasouhi, Ahmad Ghoochani

**Affiliations:** ^1^Toxicological Research Center, Loghman-Hakim Hospital, Department of Clinical Toxicology, Faculty of Medicine, Shahid Beheshti University of Medical Sciences, Kamali Avenue, South Karegar Street, Tehran 1333635445, Iran; ^2^Department of Forensic Medicine & Toxicology, Iran University of Medical Sciences, Tehran 1445613131, Iran; ^3^Department of Aerospace, Artesh University of Medical Sciences, Tehran 1781954919, Iran

## Abstract

Acute methadone toxicity is a major public health concern in Iran. Methadone-intoxicated patients are in a great risk of recurrent or delayed respiratory arrest despite the prescription of initial doses of naloxone. This study aimed to evaluate the effectiveness of oral naltrexone in the management of acute methadone overdose in opioid-naive patients and check if it could be a substitute of continuous infusion of naloxone in maintaining adequate ventilation. In a randomized, double-blind, placebo-controlled study, a total of 54 opioid-naive patients with acute methadone toxicity were enrolled. The patients received either oral naltrexone or placebo capsules after awakening by naloxone. All patients underwent close monitoring of respiration. Frequency of respiratory depression or arrest, need for another dose of naloxone, duration of hospital stay, and adverse outcomes compared between the two groups. The incidence of respiratory depression was significantly less in those who had received naltrexone. Our results show that single oral dose of naltrexone is quite efficient in the prevention of recurrent or delayed respiratory arrest in opioid-naive methadone-intoxicated patients. It can shorten the duration of hospitalization and, as a consequence, decreased the risk of complications. Further studies are warranted before the generalization of this approach to other patient populations.

## 1. Introduction

Methadone (MTD) overdose may happen after therapeutic, recreational, or accidental use. Acute poisoning with this drug is extremely common in Iran and intentional poisoning due to the ingestion of MTD syrup has become so popular in this country within the recent years [1]. 

Methadone is a synthetic, pure, long-acting opioid-receptor agonist with a long elimination half-life (almost 48 hours) that may be prolonged up to 56 hours after overdose or repeated uses [2]. It is available in our country as syrup (a mixture of R and S forms containing 5 mg/mL of MTD) and 5, 20, and 40 mg tablets [3]. A review of the registered MTD overdoses at Loghman-Hakim Poison Center in Tehran revealed an almost 100-fold increase in this toxicity within the recent decade which is probably due to the increased prescription of MTD for treatment of opioid dependence [1, 4].

Respiratory depression of MTD may manifest late and last for hours because of its long elimination half-life [2]. Deep respiratory depression, even if short term, can cause profound hypoxia with complications including hypoxic brain injury and acute respiratory distress syndrome [5, 6]. 

Patients who develop significant respiratory depression may need treatment with naloxone; this is while naloxone has a short duration of effect in contrast to most opioids. Thus, the patients need repeated or continuous doses of naloxone to prevent respiratory depression. If the MTD-intoxicated patients do not receive sufficient doses of the antidote, they may experience respiratory arrest and death [7]. 

Naltrexone (NLTX) is a competitive long-acting pure antagonist of opioid receptors and is approved by the Food and Drug Administration for the treatment of opioid and alcohol dependence [8]. It seems that it is highly effective in preventing repeated respiratory depressions and reversing the effects of *μ* opioid agonists. In Iran, NLTX is available in the form of 50 and 25 mg oral capsules [3]. It is readily absorbed in the gastrointestinal system, and it is said that administration of 50, 100, and 150 mg NLTX capsules can block all opioid receptors for 24, 48, and 72 hours, respectively [9]. However, due to its long half-life and high affinity for the *μ* opioid receptors, it may be dangerous in opioid-dependent patients and can cause severe long-term life-threatening withdrawal syndrome, heart ischemia, arrhythmias, pulmonary edema, and even sudden death [6, 7, 10]. Of course, it is not clear if the cause of naloxone-induced pulmonary edema is the withdrawal or the opioid itself [6]. Theoretically, it seems that NLTX can prevent respiratory depression and loss of consciousness in opioid-naive patients who refer after acute opioid overdose with long-acting opioids. The aim of this study was to evaluate the efficacy of single dose of oral NLTX in managing acute MTD toxicity in such patients. 

## 2. Materials and Methods

A total of 60 MTD-intoxicated opioid-naive patients were enrolled in this double-blind, placebo-controlled, and randomized clinical trial. Patients who claimed not to be opioid-dependent and had accidentally or intentionally (suicidal or recreational) ingested MTD (based on the obtained history) and had referred to Loghman-Hakim Hospital Poison Center between May and August 2012 were included. All had signs and symptoms of acute opioid toxicity. 

Those with active liver disease and increased level of transaminases (twice the normal range), acute poisoning with complications developed before presentation (such as aspiration pneumonia, brain trauma, and acute lung injury), withdrawal syndrome after taking NLTX, acute withdrawal after the administration of naloxone on presentation, and early voluntary discharge were excluded.

History was taken from the patient him-/herself or close relatives and paramedics in unconscious cases. The diagnosis was finally confirmed in all patients by bedside urine tests. The comatose patients confirmed use of methadone after regaining consciousness. The patients' data on formulation of the ingested MTD, time elapsed between the consumption and presentation, and demographic characteristics were recorded. The patients were examined for signs of toxicity followed by pulseoximetry and arterial blood gas (ABG) analysis. 

Emergency management of the airway, breathing, and circulation as well as administration of naloxone (if necessary) were also performed for those with respiratory depression (respiratory rate below or equal to 10 per minute and/or respiratory acidosis (pH < 7.36 and pCO_2_ > 44 mmHg in ABG)). Both groups were still able to receive the current standard care without restrictions (naloxone/airway interventions) if needed. 

A bag filled with 60 balls (30 with “A” letter (NLTX group) and 30 with “B” letter (placebo group) on them) was used. On admission of each patient, the administrating physician who was blind to the study drew a ball from the bag. If the letter on the ball was A, he would give a capsule from the A box (containing 30 real 50 mg NLTX capsules) and if it was B, he would give capsule from the B box (containing 30 capsules completely similar to the NLTX capsule filled with dried powdered milk). None of the treating physicians, patients, and the trial manager knew which of the boxes contained NLTX. According to the ball and box, the patients were randomized into either A or B groups. All patients with loss of consciousness or respiratory depression were fully awake and had normal respiration after administration of naloxone in the emergency department (ED) and therefore were able to ingest the capsules. Altered level of consciousness was categorized into four groups of deep coma, unresponsiveness to pain, flaccid paralysis, brainstem reflexes and respirations absence (grade IV), unresponsiveness to pain (grade III), responds to pain but not voice (grade II), and lethargy/confusion (grade I) [11]. They were monitored for LOC, cyanosis, decreased respiratory rate, decreased ventilation based on ABG results, and decreased O_2_ saturation based on pulse-oximetry results for at least 48 hours in the hospitalization period. Arterial blood gas analyses were performed on admission to the ED and repeated afterwards, if needed.

By the end of the study, the patients who had received NLTX and those who had received the placebo were compared regarding the prevalence of respiratory depression, apnea, LOC, and duration of hospital stay. Clinical findings and data were collected by five fellowships in medical toxicology. The data was analyzed by statistical package for social sciences (SPSS) software version 17 and application of student's *t*-test, Mann-Whitney *U* test, Fisher's exact test, and Chi-Square test as indicated. A *P* value <0.05 was considered to be statistically significant. The experiment was conducted with the understanding and the consent of the human subject. Ethical approval for the study was given by Shahid Beheshti University of Medical Science Ethics Committee (no. 1391-1-113-9658). The trial was also registered with Iranian Registry of Clinical Trials (no. IRCT2012062910133N1).

## 3. Results 

Sixty patients older than 14 years of age were entered into our study. Five were excluded because of early voluntary discharge and one due to withdrawal syndrome after taking NLTX. Finally, 54 patients (27 in NLTX group and 27 in placebo group) were studied. The minimum ingested dose of MTD was 20 mg and the maximum dose was 600 mg. The time elapsed between ingestion and hospital presentation was from 1 to 24 hours. The data of the patients on presentation at ED is shown in Table 1. 

Prehospital apnea had occurred in 11 patients (20.4%).  Table 2 shows prehospital respiratory status of the patients. Twenty-four (44.4%) patients received a bolus dose of naloxone due to respiratory compromise on presentation, 10 (37%) of whom were in group A and 14 (51.9%) were in placebo group (*P* = 0.27).

 A dose of 0.4 to 4 mg of naloxone had already been administrated by the paramedics. Electrocardiography on presentation was normal in 40 patients; sinus tachycardia and QTc corrected prolongation were detected in 8 (14.8%) and 2 (3.7%) patients, respectively. 

Table 3 summarizes the patients' vital signs, respiratory status, LOC, ABG, and naloxone administration on presentation time at ED.

Table 4 shows the laboratory findings of the patients in both groups. 

Table 5 compares the patients' outcome between the groups during hospitalization. Two of our patients in placebo group underwent tracheal intubation, one due to aspiration pneumonia and one due to noncardiogenic pulmonary edema. These two patients had also needed more doses of naloxone (4 and 6 mg, resp.) at emergency department (ED) due to respiratory failure. We had no death in our study.

## 4. Discussion 

Methadone is widely prescribed for treatment of opioid dependence and pain relief [12, 13]. Recently, accidental or intentional acute MTD overdose has significantly grown and become a public health problem in many countries [14, 15]. 

Ingestion of large or repeated doses may produce significant toxicity and lead to fatal respiratory depression. Thus, most of these patients may need respiratory monitoring associated with administration of high doses of naloxone in an intensive care unit (ICU) even for several days [16]. In spite of all treatments performed, these patients may develop respiratory arrest or death mainly because of the long-term effects of MTD or its late manifestations. Some authors reported that death was frequent in overdosed children and in adult after intentional intoxication [17–19]. Although almost 80% of our patients had intentionally consumed MTD, we had no death report in our patients. 

Although aspiration pneumonia (secondary to LOC), acute lung injury, rhabdomyolysis, prolongation of QT interval, torsade de pointes dysrhythmia, seizure (secondary to hypoxia), and hypothermia have been mentioned as important complications of MTD intoxication [9, 20–23], respiratory failure is its most important fatal complication [24], and bradypnea is the best predictor of respiratory depression which responds to naloxone [25]. Once MTD-related respiratory depression is suspected, initial management should focus on airway support. Close attention should also be paid to the patient's ventilation. Bradypnea and pCO_2_ elevation in ABG are reliable signs of hypoventilation. Although pCO_2_ increased in both groups, mean CO_2_ increment was significantly higher in the placebo group. 

Naloxone is the treatment of choice in opioid-related respiratory depression [26]; however, higher initial doses (2–10 mg) and prolonged continuous IV infusions may be needed to maintain adequate ventilation in cases with respiratory arrest [27]. 

Many authors believe that delayed or recurrent respiratory depression can be expected even after a dramatic response to primary administration of naloxone because of long elimination half-life of MTD [28–30]. Thus, they recommend administration of a long-acting opioid antagonist like NLTX to maintain adequate ventilation. Interestingly, all of the patients who received a single dose of 100 mg NLTX had a normal respiration during their hospitalization.

Tables 1 and 2 show that in prehospital setting no significant differences existed in the baseline characteristics of the two groups. In prehospital stage, 11 patients (almost 20%) of all participants experienced respiratory arrest, three of whom had two episodes of apnea that had been reversed by naloxone administration by paramedics. Respiratory failure was also detected in nearly half of all patients on presentation or during stay in ED. 

Table 3 shows that received naloxone dose by case group was less than that in the control group before NLTX administration. In fact, taking naloxone in 9 (33.3%) patients of the NLTX group versus 10 (37%) in the placebo group revealed that this difference was due to large doses of naloxone (more than 4 mg) for two ill cases in the placebo group. The significant difference in blood pH is also due to significant decrease in pH of these two ill patients. 

In this study, acute MTD complications significantly occurred less in the patients who had received NLTX. Nearly one third of the patients experienced respiratory depression during hospital stay. Interestingly, all of these patients belonged to the placebo group that underwent standard treatments. Theoretically, patients with acute long-acting toxicity will need continuous infusion of naloxone for prevention of recurrent apnea in spite of receiving enough initial doses of naloxone. Also, these patients need close monitoring of the vital signs preferably in the ICU. Lack of enough ICU beds, pump infusion, and other monitoring systems as well as agitation in a suicidal patient could be the main causes of not receiving enough naloxone infusions and development of sudden, life-threatening respiratory depression in them. This study showed that NLTX could effectively prevent this complication. 

Also, 10% of all participants who were admitted to our poison center re-experienced apnea and all of them were from the placebo group. Thus, this study shows that respiratory depression was significantly less in the NLTX group. 

Almost 40% of our patients in the placebo group experienced LOC during hospitalization. Significant LOC can lead to aspiration pneumonia and prolonged hospitalization time in these patients. No patient in NLTX group developed such complication. Aspiration pneumonia and ARDS happened in two patients of the placebo group and none of the patients in NLTX group. 

Laboratory results were also different between the two groups. Abnormal SGOT and CPK may reflect acute musculoskeletal effects of MTD intoxication which led to rhabdomyolysis in some patients, a condition previously demonstrated in opioid overdose and defined as the most common cause of poisoning-induced rhabdomyolysis in Iranian intoxicated patients [4, 31]. Hyperglycemia was also more prominent in placebo group and even high levels up to 319 mg/dL, supposed to be related to diabetes, were detected in these patients. Ketotic or nonketotic hyperglycemic coma has already been attributed to MTD overdose and its related deaths [32, 33]. Therefore, high blood sugar may be an indicator of severe toxicity, a sign that was controlled in NLTX group. Further investigation is needed to confirm such association. 

Patients who received NLTX did not experience respiratory depression and LOC and did not need ICU care support while those in the placebo group needed continuous administration of naloxone. On the other hand, 13 (24.1%) of total 54 patients needed antidote therapy with naloxone (bolus dose associated with IV infusion for at least 10 hours), almost all of whom were from the placebo group. Only one patient in the NLTX group needed a single bolus dose of IV naloxone (0.4 mg) because of hypercapnia (pCO_2_ > 45). However, this patient had no sign and symptom of respiratory depression. The most important complication of MTD overdose is respiratory arrest, especially in patients who do not receive continuous IV naloxone. This research shows that apnea can be prevented if opioid-naive patient takes NLTX. One of our patients in group A showed acute withdrawal syndrome and was excluded from the study although he had claimed to be a nondependant to opioids. This accidentally precipitated withdrawal syndrome suggests the use of naloxone before NLTX administration when its safety is doubted even with the patients who claim not to be dependent.

Hospital stay was significantly less in the NLTX group, and this means that prescription of NLTX could prevent important MTD-related complications such as aspiration pneumonia secondary to LOC. It also seems that it is possible to discharge asymptomatic patients with mild to moderate MTD toxicity after prescribing a single 50 mg dose of NLTX. 

Although naloxone can lead to withdrawal syndrome or acute lung injury in an opioid-dependent patient, it seems to be safe in naive patients. Therefore, NLTX should be given to those already awakened by naloxone because they are not expected to develop a withdrawal syndrome. 

Some authors believe that in most cases of intentional MTD intoxication the patient should be observed at least for 48 hours [34]. After this time, he/she may be discharged once respiration and mental status are normal and naloxone has to be stopped for at least 6 hours before disposition. Current study shows that these patients can be discharged sooner if NLTX is administered to them. We, however, had to observe our patients in NLTX group to be sure that unwanted complications would not happen. None of our patients in the NLTX group had bradypnea or respiratory arrest during hospitalization. 

Patients who ingest large amounts of MTD need to be observed for at least 48 hours; they may need readministration of naloxone and must be under close monitoring of respiration preferably in ICU. This study shows that administration of single dose of NLTX (50 mg) can prevent respiratory compromise and patients can be safely managed in ward. Opioid-naive patients with acute intentional MTD overdose can be given a dose of NLTX without worrying about withdrawal syndrome. Although we had no major complication in our survey, theoretically, MTD half-life may be prolonged in intoxicated patients exceeding the NLTX half-life. Thus, we strongly recommend another re-enforcement dose of NLTX at discharge time.

Our study supports the Nelson et al. hypothesis suggesting that acute overdose with MTD and probably other long acting *μ* opioid agonists can be effectively managed by NLTX in opioid-naive patients [6]. 

## 5. Conclusion

Administration of oral NLTX is safe, well tolerated, and effective in preventing and treating respiratory depression/LOC-related to acute MTD overdose in opioid-naive methadone-intoxicated patients because there is not any risk for withdrawal syndrome in them. A single dose of oral NLTX can prevent recurrent or delayed life-threatening respiratory depression in these patients. It also can significantly decrease the length of hospital stay, need for ICU care, and costs of treatment in methadone-naive intoxicated patients. It may also be beneficial for treatment of other long-acting opioid intoxications such as codeine, meperidine, and diphenoxylate. Further prospective investigations with more sample sizes are warranted before the generalization of this approach to other patient populations. 

## Figures and Tables

**Table 1:** Baseline characteristics of groups NLTX and Placebo.

Characteristics	Total	NLTX	Placebo	*P* value
		*n* = 27	*n* = 27	
Age				
Mean ± SD	—	26 ± 11	31 ± 12	0.03^‡^
Sex				
Male	24 (44.4%)	9 (33.3%)	15 (55.6%)	0.10*
Female	30 (55.6%)	18 (66.7%)	12 (44.4%)	
Mode of toxicity				
Suicidal	45 (83.3%)	24 (88.9%)	21 (77.8%)	0.48**
Accidental	9 (16.7%)	3 (11.1%)	6 (22.2%)	
Formulation				
Tablet	20 (37.0%)	10 (37.0%)	10 (37.0%)	>0.99*
Syrup	34 (63.0%)	17 (63.0%)	17 (63.0%)	
Ingested MTD doses (mg)				
Mean ± SD	130 ± 116	153 ± 142	106 ± 75	0.41^‡^
Time since ingestion (hrs)				
Mean ± SD	6 ± 6	6 ± 5	6 ± 6	0.34^‡^

^†^Student's *t*-test. ^‡^Mann-Whitney *U* test. *Chi-Square test. **Fisher's exact test.

**Table 2:** The prehospital status of patients.

Characteristics	Total	NLTX	Placebo	*P* value
		*n* = 27	*n* = 27	
Pre-hospital Apnea				
No apnea	43 (79.6%)	23 (85.2%)	20 (74.1%)	0.38^‡^
One episode	8 (14.8%)	2 (7.4%)	6 (22.2%)	
Two episodes	3 (5.6%)	2 (7.4%)	1 (3.7%)	
Bradypnea	27 (50.0%)	11 (40.7%)	16 (59.3%)	0.17*
Naloxone doses (mg)				
Mean ± SD	1.5 ± 0.8	1.5 ± 0.7	1.5 ± 1	0.53^‡^
Symptoms				
Nausea & vomiting	23 (42.6%)	13 (48.1%)	10 (37.0%)	0.41*
Dizziness	14 (25.9%)	7 (25.9%)	7 (25.9%)	>0.99*
Headache	3 (5.6%)	1 (3.7%)	2 (7.4%)	>0.99**
Seizure	3 (5.6%)	2 (7.4%)	1 (3.7%)	>0.99**
Weakness	5 (9.3%)	3 (11.1%)	2 (7.4%)	>0.99**
Fall down	2 (3.7%)	0 (0.0%)	2 (7.4%)	0.49**
Pruritus	2 (3.7%)	0 (0.0%)	2 (7.4%)	0.49**

^†^Student's *t*-test. ^‡^Mann-Whitney *U* test. *Chi-Square test. **Fisher's exact test.

**Table 3:** Patients clinical finding on arrival time (at ED).

Characteristics	Total	NLTX	Placebo	*P* value
		*n* = 27	*n* = 27	
Loss of Consciousness				
Nil	28 (51.9%)	16 (59.3%)	12 (44.4%)	0.15^‡^
Grade I	17 (31.5%)	9 (33.3%)	8 (29.6%)	
Grade II	6 (11.1%)	1 (3.7%)	5 (18.5%)	
Grade III	1 (1.9%)	0 (0.0%)	1 (3.7%)	
Grade IV	2 (3.7%)	1 (3.7%)	1 (3.7%)	
Heart rate (Beat/min)				
Mean ± SD	84 ± 14	85 ± 12	83 ± 17	0.71^†^
Systolic BP (mmHg)				
Mean ± SD	114 ± 15	112 ± 13	117 ± 16	0.26^†^
Diastolic BP (mmHg)				
Mean ± SD	74 ± 9	73 ± 8	74 ± 11	0.47^†^
Temperature (°C)				
Mean ± SD	37 ± 0.2	36.9 ± 0.3	37 ± 0.1	0.40^†^
Pupil size				
Normal	12 (22.2%)	7 (25.9%)	5 (18.5%)	0.51**
Miosis	42 (77.8%)	20 (74.1%)	22 (81.5%)	
Mydriasis	1 (1.9%)	0 (0.0%)	1 (3.7%)	
Dyspnea	21 (38.9%)	11 (40.7%)	10 (37.0%)	0.78*
Cyanosis	13 (24.1%)	7 (25.9%)	6 (22.2%)	>0.99*
Bradypnea	25 (46.3%)	9 (33.3%)	10 (37%)	0.79*
Tachypnea (RR > 32/min)	2 (3.7%)	0 (0.0%)	2 (7.4%)	0.49**
Apnea occurrence	8 (14.8%)	3 (11.1%)	5 (18.5%)	0.70**
Respiratory depression	21 (38.9%)	9 (33.3%)	12 (44.4%)	0.40*
Taking naloxone	19 (35.2%)	9 (33.3%)	10 (37.0%)	0.78*
Naloxone doses (mg)				
Mean ± SD	1.4 ± 1.5	0.6 ± 0.4	2.2 ± 1.7	<0.01^‡^
*ABG *				
pH				
Mean ± SD	7.32 ± 0.08	7.34 ± 0.07	7.3 ± 0.09	0.05^†^
pCO_2 _(mmHg)				
Mean ± SD	53 ± 10	50.6 ± 9	55.3 ± 10.6	0.09^†^
HCO_3 _(mmHg)				
Mean ± SD	26.7 ± 4.2	27 ± 4.4	26.4 ± 4.1	0.61^†^
paO_2_ (mmHg)				
Mean ± SD	48.4 ± 15.2	45.4 ± 16.1	51.6 ± 13.9	0.15^†^
O_2_ Saturation (%)				
Mean ± SD	85 ± 7	89 ± 5	82 ± 7	0.13^‡^

^†^Student's *t*-test. ^‡^Mann-Whitney *U* test. ED: emergency department, ABG: arterial blood gases.

*Chi-Square test. **Fisher's exact test.

**Table 4:** The laboratory data of the patients in both naltrexone and placebo groups.

	Total		NLTX		Placebo		*P *
	Mean ± SD	Median (range)	Mean ± SD	Median (range)	Mean ± SD	Median (range)	
WBC ×10^6^ *µ*L	11.5 ± 3.9	11.0 (34 to 21.7)	10.6 ± 34	10.1 (34 to 17)	12.3 ± 42	11.4 (65 to 2.17)	0.12^†^
RBC ×10^6^ *µ*L	4.62 ± 0.48	4.56 (3.87 to 5.9)	4.68 ± 0.49	4.62 (3.87 to 5.52)	4.56 ± 0.49	4.51 (3.91 to 5.9)	0.40^†^
Hb (mg/dL)	13.1 ± 2.6	13.4 (1.3 to 16.6)	13.7 ± 1.9	14.1 (9.1 to 16.2)	12.5 ± 3.1	12.4 (1.3 to 16.6)	0.10^†^
Hct (%)	40.4 ± 4.6	39 (30.1 to 49.8)	40.8 ± 5	40.3 (30.1 to 48.4)	40 ± 4.3	39 (34 to 49.8)	0.64^‡^
Platelet ×10^3^ *µ*L	225 ± 89	225 (46 to 599)	233 ± 100	232 (97 to 599)	217 ± 79	220 (46 to 396)	0.53^†^
Blood sugar (mg/dL)	117 ± 59	106 (50 to 317)	101 ± 41	91 (53 to 197)	133 ± 70	118 (50 to 317)	0.03^‡^
Blood urea (mg/dL)	27 ± 12	24 (15 to 69)	25 ± 8	24 (15 to 47)	29 ± 14	26 (15 to 69)	0.35^‡^
Creatinine (mg/dL)	1 ± 0.4	0.9 (0.6 to 3)	0.9 ± 0.2	0.9 (0.6 to 1.5)	1.1 ± 0.5	0.9 (0.6 to 3)	0.13^‡^
SGOT (U/L)	32 ± 37	22 (11 to 241)	22 ± 11	18 (12 to 63)	41 ± 49	25 (11 to 241)	0.04^‡^
SGPT (U/L)	28 ± 31	18 (5 to 167)	24 ± 22	16 (10 to 105)	32 ± 38	21 (5 to 167)	0.58^‡^
LDH (U/L)	519 ± 394	422 (216 to 2780)	526 ± 514	416 (290 to 2780)	514 ± 243	422 (216 to 1324)	0.26^‡^
CPK (U/L)	387 ± 914	110 (50 to 4450)	105 ± 37	99 (59 to 170)	656 ± 1231	144 (50 to 4450)	0.04^†^
ALP (U/L)	180 ± 61	175 (11 to 320)	177 ± 53	178 (98 to 310)	182 ± 69	172 (11 to 320)	0.80^†^
Na (mEq/L)	163 ± 169	139 (132 to 1346)	187 ± 241	139 (132 to 1346)	140 ± 3	140 (136 to 147)	0.73^‡^
K (mEq/L)	4 ± 0.4	4 (3.4 to 4.8)	4 ± 0.4	4 (3.4 to 4.6)	4.1 ± 0.4	4.1 (3.4 to 4.8)	0.17^†^
Bili. T (mg/dL)	0.6 ± 0.3	0.5 (0 to 1.4)	0.6 ± 0.3	0.5 (0 to 1.4)	0.6 ± 0.3	0.5 (0 to 1.4)	0.62^‡^
Bili. D (mg/dL)	0.2 ± 0.1	0.2 (0.1 to 1)	0.2 ± 0.2	0.2 (0.1 to 1)	0.2 ± 0.1	0.1 (0.1 to 0.3)	0.02^‡^

^†^Based on Student's *t*-test. ^‡^Based on Mann-Whitney *U* test.

**Table 5:** Comparison of patients' clinical status during hospitalization.

Outcome	Total	NLTX	Placebo	*P* value
		*n* = 27	*n* = 27	
Abnormality				
Loss of consciousness	10 (18.5%)	0 (0.0%)	10 (37.0%)	<0.01*
Bradypnea	11 (20.4%)	0 (0.0%)	11 (40.7%)	<0.01*
Apnea	5 (9.3%)	0 (0.0%)	5 (18.5%)	0.02*
ABG				
Respiratory acidosis & hypercapnia	8 (14.8%)	1 (3.7%)	7 (25.9%)	0.05**
Hypoxia	9 (16.7%)	1 (3.7%)	8 (29.6%)	0.02**
Taking naloxone bolus	13 (24.1%)	1 (3.7%)	12 (44.4%)	<0.01*
*Other clinical status *				
Naloxone iv Infusion	10 (18.5%)	0 (0.0%)	10 (37.0%)	<0.01*
Need for ICU care	14 (25.9%)	0 (0.0%)	14 (51.9%)	<0.01*
Hospital staying (hrs)				
Mean ± SD	32 ± 20	26 ± 17	38 ± 21	0.009^‡^
Median (range)	24 (12 to 96)	20 (14 to 96)	32 (12 to 96)	

*Based on Chi-Square test. **Based on Fisher's exact test. ^‡^Based on Mann-Whitney *U* test.
